# Efficacy of Limbal-conjunctival Autograft Surgery with Stem Cells in Pterygium Treatment

**DOI:** 10.4103/0974-9233.58417

**Published:** 2009

**Authors:** Walid M Abdalla

**Affiliations:** Department of Ophthalmology, Magrabi Eye and Ear Center, Muscat, Sultanate of Oman

**Keywords:** Conjunctival Graft, Pterygium, Stem Cells

## Abstract

**Purpose:**

To determine the efficacy of limbal-conjunctival autograft surgery with stem cells in the management of primary and recurrent pterygium and determine the best corrected visual acuity after surgery.

**Materials and Methods:**

Surgical excision of pterygium and limbal-conjunctival transplantation with stem cells was of 40 eyes (of 31 patients) with pterygium. Thirty one cases were primary and nine cases were recurrent pterygia. Graft margins were secured to the recipient site while stem cells aspect was sutured to the limbus.

**Results:**

After one year of follow-up, 37 of 40 (92.5%) eyes were free of recurrence. One of the three recurrent cases was aggressive (recurrence occurred two months after surgery) and the other two showed 2 mm corneal extension at 12 months follow-up. In 24 patients, out of 40 (60%), best corrected visual acuity improved more than two lines.

**Conclusion:**

Limbal-conjunctival autograft surgery, including stem cells, appears to be an effective surgical technique in preventing pterygium recurrence and it can also help in improving the best corrected visual acuity.

## INTRODUCTION

Pterygium is a common external eye disease, seen more frequently in tropical and subtropical areas where exposure to ultraviolet sunlight is high. The main histopathological change in primary pterygium is elastodysplasia and elastodystrophy of subepithelial connective tissue.^1^ Indications for surgical excision include impending or manifest visual loss due to involvement of the central cornea, irregular astigmatism, restriction of ocular motility and atypical appearance leading to concerns of squamous neoplasia.^2^ Surgical treatment of pterygium is directed at excision, prevention of recurrence and restoration of ocular surface integrity.

The main concern of simple excision of pterygium is the high recurrence rate.^3^,^4^ To prevent recurrence, adjunctive therapies are to be considered. These include application of antimetabolites such as mitomycin C, radio- therapy, conjunctival or limbal conjunctival autograft^5^ and amniotic membrane graft. Unacceptable recurrence rates led to the abandonment of excision with bare sclera technique.^6^ There is widespread acceptance of conjunctival autografting, since its introduction by Thoft in 1977 and application to pterygium by Vastine *et al*. and Kenyon *et al*.^5^ However, no single autograft technique is completely effective in preventing recurrence. Most reports also advocate a thin graft devoid of Tenon's fascia but one which is large enough to completely cover the bare scleral defect.^5^

Limbal conjuctival autograft, using stem cells, is reported to be an effective adjuvant to lower the recurrence rate of pterygium. This study was carried out to determine the long term recurrence rate, visual acuity improvement and astigmatic changes after excision of pterygium and conjunctival autografting using limbal stem cells.

## MATERIALS AND METHODS

The technique used was excision of pterygium, extending at least 2 mm beyond the limbus, followed by superior conjunctival limbal autograft including limbal stem cells performed in 40 eyes of 31 patients between February and October 2007 at our institution. Thirty one eyes had a diagnosis of primary pterygium and nine were recurrent cases. Patients with other ocular surface disease or ocular pathology were excluded from the study. None of the patients had previously undergone any ocular procedure. Informed consent was obtained from all patients. Institutional review board and ethics committee approval was not required.The surgical technique used was based on that described by Kenyon *et al*. who reported that the harvesting of the conjunctival graft should not stop at the limbus but continued into clear cornea for about 2 mm to harvest limbal stem cells. Local anesthesia was administered using Van Lint and peribulber injection of 50% Marcaine (0.75%) and 50% Xylocaine (2%) without epinephrine. Westcott scissors were used to excise the body of the pterygium 5 mm posterior to the limbus during which care was taken to identify the insertion of the adjacent medial rectus muscle. The dissection was done down to bare sclera. And extended anteriorly to the limbus where the head of the pterygium was separated from the corneal epithelium 2 mm anterior to the pterygium head. The corneal defect was shaved for any residual tissue using a blade. Any bleeding points were cauterized with wet field diathermy. The size of the conjunctival graft required to resurface the exposed scleral surface was determined by Castroviejo calipers by taking measurements that covered the area of defect created by excision. The measured dimensions were used to determine the exact size of the graft from the superior temporal bulbar conjunctiva using a marking pen. A non toothed forceps was used to rotate the globe. Stay sutures and injection of balanced salt solution to separate the conjunctival graft from the underlying tenon's capsule were avoided. Care was taken to obtain thin conjunctiva without buttonholes, the graft was then continually dissected until the limbus was reached using sharp blade till clear cornea was seen 2 mm from the anatomical limbus. Westcott scissors were used to separate the conjunctivo limbal graft and the free graft was rotated and moved to the scleral bed maintaining limbus to limbus orientation. The graft was secured with interrupted 8–0 vicryl sutures. The donor site was left to epithelialize without closure of the defect. A bandage contact lens was applied post operatively for all patients for one week.

After surgery, steroids, antibiotics and artificial tear drops were used four times daily for four weeks. Demographic, preoperative, operative and post operative details including complications were obtained from the case notes of the patients. These patients were invited for clinical review carried out by the same surgeon. During the review; best corrected visual acuity, refractive error, slit lamp findings were noted. Attention was given note if there was recurrence of pterygium and other complications were also noted. Recurrence of pterygium was defined as a fibro-vascular in-growth of 1.5 mm or more beyond the limbus with conjunctival drag as used by Singh *et al*.^4^ Photographs were taken in all patients who attended the review appointments.

## RESULTS

Of the 40 operated eyes, 28 were reviewed at a mean follow-up period of 13.5 months (range 12-15 months). Data collected from those patients is reported in Table 1.

**Table 1:** Demographic data of patients undergoing limbal-conjunctival autograft surgery with stem cells in the treatment of pterygium

Number of eyes (patients)	40 (32)
Laterality (Right:Left)	19:21
Male:Female	22:18
Age (years)	21:57
Follow up periods (months)	12:15
Pterygium (Primary:Recurrent)	31:9

These patients were seen on the first post operative day, at one week, two weeks and one month post-operatively and at the time of final follow-up. All patients were Omani, 22 male and* female.In all eyes, pterygium was located nasally. All the eyes were operated by the same surgeon. Three patient required general anesthesia whereas the rest were operated on using peribulber anesthesia. In cases where the pterygium was bilateral, each eye was analyzed separately. Improvement in best spectacle corrected visual acuity was seen (two to six Snellen lines) in 24 patients. Recurrence was seen in three eyes (7.5%). The first, patient, a female, in her early twenties had a previously recurrent pterygium which developed following simple excision of the pterygium, one year prior to enrolment. The recurrence in this patient was noted three months post-surgery. The other two patients were men who had primary pterygia and both had a small fibro vascular band that extended 2 mm anterior to the limbus and were first noticed one year after surgery.

Peripheral corneal scarring at the site of the pterygium occurred in four patients and dellen formation developed in one patient. Symblepharon formation or severe conjunctival scaring was not observed at the donor site in any of the patients [Table 2].

**Table 2:** Complications of limbal-conjunctival autograft with stem cells

Complications of limbal-conjunctival autograft with stem cells	Number of cases	Percentage
Recurrence	3	7.5
Corneal scarring	4	10
Dellen former	1	2.5

## DISCUSSION

Simple excision of pterygium is associated with a high recurrence rate ranging from 30 to 70%,^3^,^4^ To reduce this high recurrence rate, different methods like, beta irradiation, mitomycin C, and amniotic membrane have been used.^4^,^11^ However, serious complications such as secondary glaucoma, uveitis, scleromalacia and corneal perforation are associated with these methods.^7^ Contamination of amniotic membrane is a potential risk that cannot be overlooked despite of low recurrence rate^8^.

Pterygium excision followed by conjunctival autograft is associated with recurrence rate of 5.3 to 39%.^5^

After the initial report by Kenyon *et al*.,describing the success of conjunctival autografting following pterygium excision, other authors have largely failed to achieve the same success rate^4^,^5^. The wide range of recurrence rates reported number of factors. Review of published literature suggests that the surgical technique could probably be the single most important factor influencing recurrence. The meticulousness with which the limbal tissue is included in the autograft, in our opinion, determines the success of the procedure.

Various studies have specifically described the inclusion of limbal tissue in the graft and have demonstrated low recurrence rates. 5,9 The importance of limbal transplantation in ensuring low recurrence rates has also been stressed by Figueiredo *et al*., 10 but their work was carried out on a higher mean age group than the mean age in this study.

Minimal limbal conjunctival autograft showed a recurrence rate of 9.2% during a followup period of 6–29 months.11 In our study the recurrent rate of 7.59% was comparable in a followup period of 12–15 months. Using other procedures others have shown varying degrees of recurrence that ranged from 0–15%.^11^,^12^ We demonstrated improvement in astigmatic correction in 60% of our patients which is slightly lower than that demonstrated by Oguz and colleagues who showed that 75% of patients had improvement in the astigmatism after prteygium surgery. We believe that this difference could be explained by the smaller numbers in our series.

A major drawback for limbal- conjunctival autograft transplantation is that it is technically more demanding and time-consuming. Hence we conducted this study on limbal conjunctival autograft as an effective procedure in treating pterygium. The main limitation that demonstrated of this study is the small number of patients and lost patients in follow-up despite multiple attempts to trace and call them for review. In conclusion, limbal-conjunctival autograft appears to be an effective conjunct technique in preventing pterygium recurrence and can also help to improve the best corrected visual acuity of patients.
